# Knowledge, attitude and practice of smoking cessation counseling among dental hygienists in Saudi Arabia

**DOI:** 10.18332/tpc/145530

**Published:** 2022-02-25

**Authors:** Areej J. Alsiwat, Haya M. Alayadi

**Affiliations:** 1 Dental Biomaterials Research Chair, Dental Health Department, College of Applied Medical Sciences, King Saud University, Riyadh, Saudi Arabia

**Keywords:** smoking cessation, dental hygiene, counselling

## Abstract

**INTRODUCTION:**

Tobacco has major effects on the oral health of an individual. Dental hygienists play an important role in smoking control by educating and motivating patients and community. The aim of this study is to add more evidence on knowledge, attitude, and practice of smoking cessation counseling among dental hygienists in Saudi Arabia.

**METHODS:**

The study is a cross-sectional study, subjects are dental hygienist practitioners in Saudi Arabia employed in either public or private hospitals in 2021. The data collection tool of this study was a semi-structured self-administered questionnaire of 36 questions. The final sample included 80 employed participants.

**RESULTS:**

Knowledge differed significantly amongst participants according to their educational level, with the highest mean knowledge scores among PhD versus Bachelor’s degree holders [mean (SD) = 27.7 (29.3) vs 21.9 (10.8), p=0.038, respectively]. On the other hand, dental hygienists attitude percentage scores differ statistically by working place; dental hygienists working in public health organization had a score [mean = 74 (8.9)] was higher compared to those in private health organizations [mean = 69.3 (10.8)]. Also, participants attitude percentage mean scores differ statistically by years of experience; the higher the years of experience the higher the dental hygienists’ adaptation of an attitude toward smoking cessation counselling. In terms of dental hygienists practice percentage mean scores, the only statistically significant difference was educational level [Bachelor’s: 64 (17.7); Master’s: 103.3 (17.2); PhD: 108.8 (15.3); p=0.003].

**CONCLUSIONS:**

Dental hygiene practitioners’ level of knowledge is unsatisfactory. Workshops and program training are indeed necessary to increase dental hygiene knowledge leading to effective implementation of smoking cessation counselling.

## INTRODUCTION

Tobacco has been a worldwide public health threat and a cause of avoidable morbidity and mortality. More than 8 million people a year die as a consequence of tobacco-related diseases^1^. Tobacco is not only a risk factor for many diseases that affect overall health, such as cancers of the head, neck, throat, esophagus, but also has significant regional effects on the oral health of an individual, including cancer of the mouth, tongue, lips and gums, as well as various dental diseases^1^.

Because cigarette smoking involves exposure to chemical compounds, it is an active player in the pathogenesis of periodontitis. Although the relation between smoking and periodontitis is well-supported by clinical studies, unfortunately, the precise biological mechanisms related to this association have not been defined yet^2^. Therefore, to date, smoking cessation has been emphasized as the best practicable way to decrease the risk of the onset and progression of periodontitis and to improve treatment outcomes.

Primary healthcare providers have the opportunity to help smokers to quit. Dental hygienists play an important role as primary healthcare providers. However, a previous study reported that 64% of general dental practitioners lacked training on smoking cessation during their undergraduate training^3^. Furthermore, lack of time, the assumption that smoker patients have no motivation to quit, and the lack of confidence in their knowledge and skills in smoking cessation counselling have been reported as main barriers^4,5^. In addition, when asked about their knowledge and practice, more than 50% of family physicians requested training to improve their skills in smoking cessation counselling^6^.

Saudi Arabia is one of the top ten countries that consume cigarettes with a 12.1% population prevalence of cigarette smoking^7^. However, there seems to be a lack of national studies that assess the knowledge, attitude and practice (KAP) of smoking cessation counselling. Thus, this study aims to assess the knowledge, attitudes, and practice of smoking cessation counselling among dental hygienists in Saudi Arabia.

## METHODS

### Study design

The study is a cross-sectional study conducted in Saudi Arabia between May 2021 to June 2021. The study subjects were dental hygienist practitioners in Saudi Arabia employed in either public or private hospitals.

Saudi Arabia has three colleges providing a Bachler’s Degree in Dental Hygiene. The colleges provided the list of graduates, and then an email was sent to all graduates. We sent an invitation to 150, while the final response rate was 73.3 %. Based on a study by Joufi et al.^8^, the estimated number of licensed dental hygienists in Saudi Arabia was around 298; we aimed to target 100 participants in our study. Out of the 139 responses, 110 completed the questionnaire, to the end there was a degree of missing questions. We excluded only responses with incomplete demographics. While mean imputation was performed to missing numerical data, deterministic regression imputation was performed to missing categorical data. Thirty (n=30) of the participants were non-employed and as the study purpose was to assess the practice of smoking cessation counselling, we excluded these non-employed from the analysis. Thus, the analysis was conducted among 80 employed participants.

### Data collection tools

The data collection tool of this study was a semi-structured self-administered questionnaire of 36 questions^9^. Permission was obtained from the questionnaire developers. The questionnaire includes three domains; it begins with socioeconomic data, then questions related to the assessment of Knowledge, Attitude and Practice about smoking cessation counselling, and the final section includes questions about the barriers to smoking cessation counselling. The questionnaire was administered in English to the study sample, but minor language modifications were applied, thus face validity was assessed by two external researchers. A pilot study was also conducted on 20 dental hygiene staff from King Saud University. The interrater reliability was 0.84.

### Data scoring

Data were handled in the Knowledge, Attitude and Practice section as follows; a score of ‘1’ was assigned to a correct response in the Knowledge section while a ‘0’ score was assigned to a wrong response. The total knowledge score was generated by adding up the score of knowledge response, which included 12 questions (0–12 score). The overall knowledge mean and percent score was calculated from this score.

In the Attitude section, responses were based on a 5-point score Likert scale ranging from 5=‘strongly agree’ to 1=‘strongly disagree’. The total attitude score was obtained through the sum of 7 attitude section questions. Thus, the overall attitude score ranged 7–35. This was followed by calculating the attitude mean score in addition to the overall attitude percent score, which was calculated from the overall attitude score. Another variable was created to present agreement and disagreement of the attitude toward counseling on smoking cessation by adding up ‘agree’ and ‘strongly agree’ to generate an ‘agree score’ and adding up ‘disagree’ and ‘strongly disagree’ to generate a ‘disagree score’. An agreement and disagreement towards the attitude of counselling percent score was calculated from these two scores.

The Practice section included four responses. A score of ‘3’ was assigned for ‘always’, ‘2’ was assigned to ‘sometimes’, ‘1’ was assigned for ‘rarely’ and ‘0’ was assigned for ‘never’. This would indicate a score between 0–30 to the practice section as it includes ten questions. An overall practice mean and percent score was calculated from this score.

The questionnaire was conducted online through the Survs website. Data were managed and analyzed using the Statistical Package for Social Science (IBM SPSS for Mac version 26). Descriptive analyses were performed. Frequencies and percentages were calculated for categorical variables, and mean and standard deviation were calculated for continuous numerical variables, in addition to testing the statistically significant difference of KAP regarding demographic data. The chi-squared and t-tests were used for categorical and continuous numerical variables, respectively. The difference between average score means compared to the main factor (education level and years of experience) was also assessed through one-way ANOVA, based on the normality of the variables. A stepwise linear assessment model was utilized to predict the outcomes according to the different factors.

## RESULTS

The study assessed the knowledge, attitude, and practice of smoking cessation counselling among dental hygienists in Saudi Arabia. The sample included 110 participants; 30% males and 70% females. Most of the sample graduated from King Saud University (60%), followed by other international universities (32%). Detailed demographics are presented in Table 1.

**Table 1:** Sociodemographic characteristics of dental hygienists in Saudi Arabia, 2021 (N=110)

*Characteristics*	*n (%)*
**Age** (years)	
<30	75 (68.2)
30 to <40	24 (21.8)
40 to <50	7 (6.4)
≥50	4 (3.6)
**Gender**	
Male	34 (30.9)
Female	76 (69.1)
**Graduated university**	
King Saud	65 (59.1)
King Abdulaziz	3 (2.7)
Riyadh Al Elm	6 (5.5)
Other international	36 (32.7)
**Educational level**	
Diploma	6 (5.5)
Bachelor’s	89 (80.9)
Master’s	10 (9.1)
PhD	5 (4.5)
**Ranking in Saudi commission of health specialties**	
Specialist	89 (80.9)
Senior specialist	17 (15.5)
Consultant	4 (3.6)
**Years of experience**	
1–5	27 (24.5)
6–10	55 (50.0)
11–15	11 (10.0)
≥16	7 (6.4)
**Current employer** (health organization)	
Private	29 (26.4)
Public	51 (46.4)
Non-employed	30 (27.3)
**Smoking status**	
Smoker	20 (18.2)
Non-smoker	90 (81.8)
**Smoking frequency** (cigarettes per day)	
Occasionally	12 (83.7)
<5	4 (8.2)
5–9	1 (2.0)
10–14	1 (2.0)
15–24	1 (2.0)
≥25	1 (2.0)

**Figure 1:**
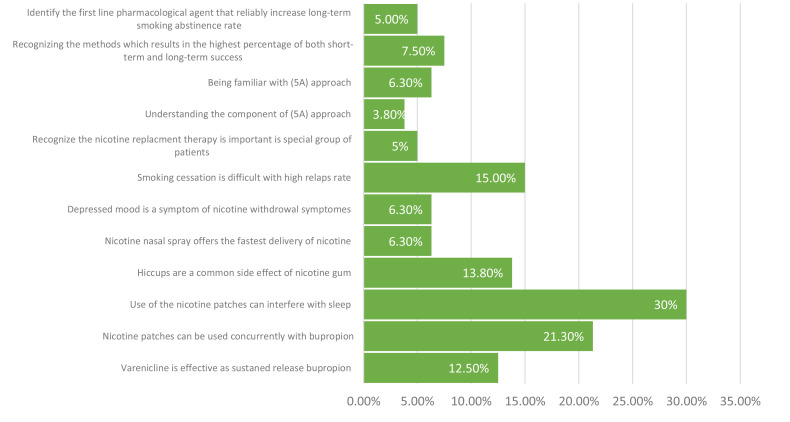
Knowledge of dental hygienist practitioners toward smoking cessation counselling (n=80)

**Figure 2:**
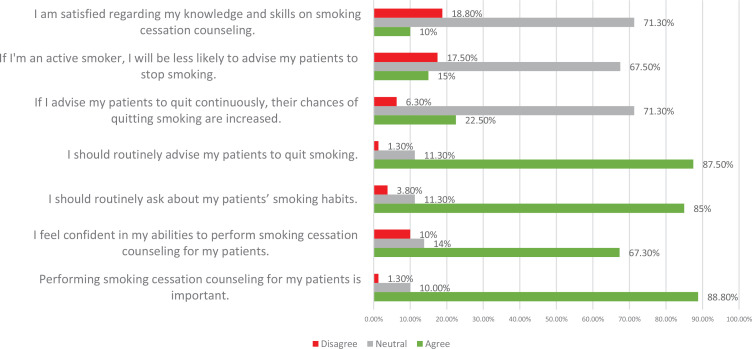
Attitude of dental hygienist practitioners toward smoking cessation counselling (n=80)

Figure 1 presents the percentage of participants who responded correctly in the knowledge section. Generally, less than half of the participants responded correctly to all questions of the knowledge section; 30% of respondents answered correctly to the question regarding nicotine pastiches interfering with sleep. Only 3.9% responded correctly to the question regarding understanding the components of the 5A approach. The participants’ attitude toward counselling regarding smoking cessation is shown in Figure 2. Only 10% of the participants were satisfied with their knowledge regarding smoking cessation counselling. On the other hand, around 88% of the participants acknowledge the importance of smoking cessation.

The participants practice score in smoking cessation counselling indicates that most participants performed smoking cessation counselling in aspects of ‘to ask’ and ‘to document’ (28.7% and 22.5%, respectively). While there were aspects rarely performed as ‘to teach’ and ‘to discuss the barriers’ (both 20%), there were aspects that were rarely performed as ‘making a plan’ (18%).

Overall, participants’ percentage mean scores (SD) were 22.6% (SD=11.6) for knowledge, 72.3% (SD=8.4) for attitude, and 90% (SD=16.5) for practice.

Table 2 presents the difference of the participants in knowledge, attitude and practice based on their sociodemographic characteristics. Knowledge mean score differed significantly amongst participants according to their educational level, with the highest mean knowledge scores among PhD and lowest among Bachler’s degree holders [27.7 (29.3) vs 21.9 (10.8), p=0.038, respectively]. However, there was no statistically significant difference in participant knowledge percentage score by age, gender, university attended, rank according to the Saudi Commission of health speciality, years of experience, place of work, or smoking status. On the other hand, the dental hygienists attitude percentage scores differed statistically by working place, dental hygienists working in public health organization with mean 74 (8.9) were higher compared to those working in private health organization with mean 69.3 (10.8). Also, participants’ attitude percentage mean scores differed statistically by years of experience; the trend suggests that the higher the years of experience, the higher the adaptation of an attitude toward smoking cessation counselling. In terms of dental hygienists’ practice percentage mean scores, the only statistically significant difference was according to their educational level. The higher the educational level the higher the practice percentage mean score [Bachelor’s: 64 (17.7); Master’s: 103.3 (17.2); PhD: 108.8 (15.3); p=0.003].

**Table 2:** Participants’ knowledge, attitude and practice scores according to the sociodemographic characteristics of dental hygienists (N=80), Saudi Arabia 2021

*Characteristics*	*Knowledge*	*Attitude*	*Practice*
	*Mean (SD)*	*Mean (SD)*	*Mean (SD)*
**Total**	2.97 (1.43)	25.54 (3.24)	26.98 (5.23)
**Age** (years)			
<30	2.99 (1.447)	25.04 (3.183)	26.57 (4.840)
30 to <40	3.33 (1.204)	26.62 (3.085)	28.61 (6.910)
40 to <50	2.43 (1.397)	26.16 (2.223)	25.41 (2.812)
≥50	1.50 (1.732)	27.27 (5.290)	27.49 (1.735)
**p**	0.047	0.119	0.330
**Gender**			
Male	2.62 (1.436)	25.35 (3.075)	26.84 (4.314)
Female	3.13 (1.430)	25.62 (3.326)	27.04 (5.618)
**p**	0.082	0.688	0.855
**Graduated university**			
King Saud	3.17 (1.587)	25.41 (3.405)	27.42 (5.443)
King Abdulaziz	2.67 (0.577)	26.02 (0.842)	31.32 (7.517)
Riyadh Al Elm	2.67 (1.033)	28.10 (3.797)	23.32 (6.706)
Other international	2.69 (1.191)	25.30 (2.851)	26.43 (4.101)
**p**	0.396	0.251	0.118
**Educational level**			
Bachelor’s	2.94 (1.366)	24.95 (2.811)	26.50 (4.783)
Master’s	3.10 (1.287)	27.46 (3.452)	30.49 (49 (5.130)
PhD	3.60 (2.881)	31.80 (3.563)	29.40 (11.651)
**p**	0.722	0.001	0.089
**Ranking in Saudi commission of health specialties**			
Specialist	2.94 (1.385)	25.28 (2.722)	26.49 (5.320)
Senior specialist	3.06 (1.819)	26.48 (5.269)	29.58 (4.546)
Consultant	3.25 (0.500)	27.27 (2.158)	26.74 (2.871)
**p**	0.179	0.021	0.082
**Years of experience**			
1–5	2.89 (1.474)	24.96 (2.826)	26.88 (5.155)
6–10	2.91 (0.831)	25.65 (2.915)	24.80 (5.555)
11–15	2.90 (2.025)	27.25 (3.822)	30.69 (6.480)
≥16	2.50 (1.225)	27.11 (3.862)	27.48 (1.232)
**p**	0.819	0.306	0.207
**Current employer** (health organization)			
Private	2.72 (1.556)	24.25 (3.786)	25.54 (5.426)
Public	2.92 (1.383)	25.91 (2.166)	27.83 (4.513)
**p**	0.287	0.020	0.171
**Smoking status**			
Smoker	2.75 (1.251)	35.19 (2.985)	24.63 (5.971)
Non-smoker	3.02 (1.469)	25.44 (3.301)	27.50 (4.939)
**p**	0.444	0.491	0.026

The one-way ANOVA test was used to determine the difference between the mean scores of each knowledge, attitude and practice within different educational level and years of experience categories. Education level was the most significant predictor in the attitude domain (p<0.001).

A stepwise linear assessment model was used to identify possible predictors of a higher attitude outcome out of the following candidate variables: age, gender, university attended, and smoking status. At each step, variables were chosen based on p-value, and a p-value threshold of 0.05 was used as a limit of the total number of variables included in the final model. The model was significant (F=21.03; p<0.001, R^2^=20%) and contained two steps. The final model consisted of educational level (b=0.401; p<0.001) and age (b=0.197; p=0.024) (Table 3).

**Table 3:** Stepwise linear regression results of average attitude score with related sociodemographic characteristics of dental hygienists, Saudi Arabia, 2021 (N=80)

	*Unstandardized coefficient*		*Standardized coefficient*		
	*b*	*SE*	*b*	*t*	*p*
**Model 1**					
(Constant)	2.94	0.160		18.38	<0.001
Education	0.333	0.773	0.404	4.856	<0.001
**Model 2**					
(Constant)	0.773	0.173		16.023	<0.001
Education	0.331	0.071	0.401	4.643	<0.001
Age	0.118	0.052	0.197	2.283	0.024

Dependent variable is average attitude score. R^2^ (Model 1) = 0.163; R^2^ (Model 2) = 0.202.

## DISCUSSION

Tobacco use in Saudi Arabia is a threat to oral health and brings forward both health and economic burdens^10^. Primary healthcare practitioners (PHCP) are said to be in the first line of addressing smoking cessation^11^. A study done by Aljdani et al.^12,13^ in the western region of Saudi Arabia, found that only 10% of their practitioners were smokers. Similar to this study, the results of the present study shows that only 18% of the dental hygiene practitioners smoke.

Previous studies reported that the Internet and books were the most common source of information regarding smoking cessation counselling due to the lack of educational content in the undergraduate medical curriculum. In our study, other than books and the Internet, published articles were a source of information for dental hygiene practitioners. This might indicate the need to modify the undergraduate curriculum through engaging in smoking cessation counseling. Also, it shows that continuing education is essential in increasing dental hygiene practitioners smoking cessation counselling knowledge.

A study by Aljdani et al.^11^ found that the majority of the PHCPs stated that they rarely asked their patients about their smoking or encouraged them to quit. In contrast to this study, a study done by Jannat-Khah et al.^14^ found that 90% of dental providers routinely asked their patients about tobacco use, but only 45% offered patient counselling. This was similar to the results of our study which found most of the dental hygiene practitioners asked and documented their patients smoking status but rarely taught and discussed how to quit smoking with their patients; this could be a result of their lack of knowledge on how to practice smoking cessation counselling with patients.

A study done in Al Madinah Al Munwarrah reported that (60%) of physicians had good knowledge of smoking cessation counselling. They also reported adequate practice in some areas^12^. Our study found that around 88% of the participants acknowledge the importance of smoking cessation. Yet only 10% of the participants are satisfied with their knowledge regarding smoking cessation counselling. This could be due to the lack of educational content in their undergraduate curriculum.

A study conducted in Riyadh by AlAteeq et al.^13^ found that all primary healthcare physicians stated that giving advice on smoking cessation counseling was part of their job, and the majority felt that having special clinics motivated them to use these methods. Our study assessed the difference between private and public health organizations, it showed that dental hygienists’ attitude towards smoking cessation counseling in public health organization was higher compared to those working in private health organization. To elaborate, this might be due to appointment duration per patient or perhaps the numeration system. In most of the Saudi Arabia private health organizations, the renumerations system is based on treatment services rather than preventive services, this would direct the attitude of health practitioners to the treatment services. Furthermore, the study also reported that doctors with higher educational levels, those with more experience (above 5 years) and those who were older (aged >40 years) had significantly better attitudes and good practices^13^. Similar to this study, dental hygiene practitioners with higher education level had more smoking cessation counselling knowledge and practice. In addition, more experience showed a greater attitude towards smoking cessation counselling.

The findings regarding the main barriers against smoking cessation counselling reported by almost all the previous studies were consistently the lack of training and time^11-13^. Similar to this study, lack of training was the most common barrier. In addition, the majority of dental hygiene practitioners reported their willingness to be involved in smoking cessation counselling programs. Furthermore, efforts to encourage dental care providers in tobacco prevention should require system and organization change. Previous studies found that developing and adopting formal delivery systems can lead to appropriate delivery of clinical preventive services^15,16^.

### Limitations

Limitations of this study include its cross-sectional design and limited sample size that may not lead to generalizable results to dental practitioners of other regions or the practice of other primary healthcare providers in Saudi Arabia.

## CONCLUSIONS

Although most of the dental hygienist acknowledged the importance of smoking cessation counseling, they were less satisfied with their level of knowledge and skills, which in turn influenced the delivery of smoking cessation counselling to the patients. Workshops and program trainings are necessary to increase dental hygiene knowledge leading to effective implementation of smoking cessation counselling for patients, individuals, families and the community. Furthermore, system and organization change may be needed to lead to appropriate delivery of clinical preventive services.

## Data Availability

The data supporting this research are available from the authors on reasonable request.
